# Retraction: A novel image encryption scheme based on quantum dynamical spinning and rotations

**DOI:** 10.1371/journal.pone.0338963

**Published:** 2025-12-17

**Authors:** The PLOS One Editors

The *PLOS One* Editors retract this article [1] due to concerns about:

- The use of terminology that differs from standards in the field (e.g., ‘huge information’ instead of ‘big data’, ‘great degree touchy’ instead of ‘highly sensitive,’ etc.) resulting in large parts of the text being difficult to understand.
- References to retracted work cited in References 14, 17, 18, which were retracted after [1] was published.
- Compromised peer review.

The Editors have no evidence of any author involvement in the peer review concerns.

All authors did not agree with the retraction.
